# Subungual Exostosis

**Published:** 1904-03

**Authors:** W. Roger Williams


### SUBUNGUAL EXOSTOSIS.

BY

W. ROGER WILLIAMS, F.R.C.S. Eng.

THE curious affection known as "subungual exostosis" appears to have been scientifically described for the first time by Dupuytren, who discriminated cases as early as 1817; hence it is also often called "Dupuytren's exostosis."

In studying the natural history of this condition, one cannot help noticing the great frequency of its occurrence as compared with other ossiform skeletal outgrowths; for of 111 consecutive formations of this kind tabulated by me, 35—or more than one-third—were subungual. In like manner, of 82 osteomata at Guy's Hospital from 1889 to 1901, 26 were subungual (McGarvin).

Another striking peculiarity is the almost complete limitation of the disease to the terminal phalanx of the great toe, for this was its localisation in 34 out of the 35 cases comprised in my list.

In the exceptional case alluded to, the outgrowth was connected with the terminal phalanx of the middle toe of the right foot, the patient being a girl eleven years old. Other instances of this kind have been reported by Debron and Chassaignac. Next to the great toe, the little one is most frequently affected—of which Paget, Liston, Debron, and others have met with instances. In the Hunterian Museum (Nos. 1595–8, Pathological Series) are several specimens of subungual exostoses prepared by Liston, in one of which the tumour is connected with the ungual phalanx of the little toe, near its distal extremity. They have been seen on the second toe by Gosselin and Miramond, and on the fourth toe by Cusco; in short, any of these toes may very exceptionally be affected.

Similar tumours have, in a few rare cases, been seen also on the digits (Fontanel, Gosselin, Hutchinson, etc.).

The subungual exostosis is almost invariably solitary and unilateral; but in a young woman, seventeen years old, Poncet found a symmetrical formation of this kind on each great toe.

Of 23 cases tabulated by me, in 14 the right foot was affected and in 9 the left.

These outgrowths are usually of small size, few of them being larger than a pea. Having increased slowly for some years, they then cease to grow. In this mature state they are firmly attached to the inner (tibial) side of the ungual end of the phalanx, in such a manner that the tumour appears to be a branch from the latter. On section of dried specimens, the tumour is seen to be a real outgrowth from the phalanx, consisting externally of dense cortex and internally of trabecular structure, both of which are directly continuous with the corresponding structures of the phalanx. Such is the appearance of a typical mature specimen; but between this complete blending of the normal part with the morbid structure and their complete separation, every intermediate grade may be met. Pediculated forms are of common occurrence, the extent of the osseous connection being very variable.

The connection of these outgrowths with the inner margin of the terminal end of the phalanx is a feature of great constancy; but exceptionally their attachment is to the dorsal aspect of the phalanx, or even, it is alleged, to its external margin or free end.

Examples of discontinuous formations of this kind, which are rare, have been reported by Trélat and Blandin, who compared them to sesamoid bones. In a case described by Chassaignac, a tumour of this kind was situated in a depression on the surface of the phalanx, from which however it was quite distinct.

A fact of remarkable interest about these growths is that they evolve through cartilage, and their ossification is believed to take place from but a single centre.

The subungual exostosis is usually invested externally with a thick layer of fibrillar tissue, which is continuous with the periosteum of the phalanx; while beneath this, between it and the bone, a layer of fibro-cartilage may generally be found, which is the remains of the original chondromatous rudiment.

From what has been stated, it may be inferred that subungual exostoses originate from a cartilaginous "rest," which is generally situated beneath the periosteum of the phalanx, in the vicinity whence these outgrowths commonly arise.

Here it seems desirable to call attention to the tendency which subungual exostoses have to re-form locally after removal, unless their matricular structures are completely extirpated, which, as a rule, can only be assured by removing at least the terminal portion of the phalanx together with the outgrowth. The following is a good example of the consequence of neglecting this precaution:—

A healthy-looking boy, nine years old, came under my notice with a recurrent tumour of this kind. One year previously he had first noticed a small hard tumour beneath the inner border of the nail of his left great toe. Some months later this was snipped off, but the present growth soon afterwards began to form. The recurrent disease, with a small portion of the adjacent part of the phalanx, was now excised; but evidently not enough was removed, for nine months later he was seen again with a fresh growth, the size of a pea, in the same situation as before. The histological structure of the growth was that of an ordinary subungual exostosis, capped with fibro-cartilage. This second recurrence was dealt with by removing the tumour, together with all but the articular extremity of the phalanx, and he was thus completely cured.

Here it seems desirable to point out that these little tumours are essentially benign. I am not aware of a single instance in which a growth of this kind has ever manifested malignant properties. Consequently, I am surprised to find a statement in a recently-issued *Manual of Surgical Treatment*, to the effect that these tumours "are not infrequently sarcomatous." It is true that Cornil some years ago, basing his diagnosis solely on histological grounds, described a specimen of this disease as "ossifying sarcoma"; but I believe all surgical pathologists now recognise that this was a mistake. However this may be, I have convinced myself that the ordinary subungual exostosis is a benign formation, with little or no tendency to malignancy.

These formations, as a rule, first attract notice during adolescence, the average age of 13 patients under my observation being 18 years. The first indications of the disease were generally noticeable from a few months to one year earlier than this. The earliest age at its onset was 8 years, and the latest 29: 10 out of my 13 patients were from 15 to 25 years old, and this is the usual period for its onset. Gosselin has met with an instance in a woman 47 years old. A discontinuous tumour of this kind, of congenital origin, has been described and figured by Annandale.

Both sexes are liable to be affected, although it is of much commoner occurrence in females than in males, 27 of my 35 cases being females.

In the majority of cases the disease appears to arise spontaneously; that is to say, without any obvious external cause. This was so in 10 out of 13 cases as to which I have information; in the other 3 cases there was a history of previous injury.

I know of no instances of hereditary transmission, although, on *à priori* grounds, it seems likely that such may occur.

With regard to the clinical features of the disease, the first thing to attract attention generally is pain in walking. On examination a small hard tumour, fixed to the phalanx, may be found projecting slightly beneath the inner side of the nail, which is often more or less deformed and displaced. The adjacent soft parts are apt to be eroded and swollen through inflammation. Owing to complications such as these, the condition may easily be mistaken for ingrowing toe-nail, onychia, or for a warty or corn-like growth.

As previously mentioned, the only effective treatment is to remove the tumour, together with the distal end of the phalanx whence it grows. The articular end of the phalanx and the inter-phalangeal joint should not be interfered with, as this would weaken unnecessarily the arch of the foot. An endeavour should be made to save the matrix of the nail. The operation is effected by cutting small anterior and posterior flaps, and dissecting them off the phalanx sufficiently to expose the tumour and the part of bone whence it grows, which is then cut off with a bone forceps.

In discussing the origin of subungual exostosis, it is necessary to point out at the outset, that the tumour can have no genetic relationship with the epiphyseal cartilage of the phalanx, which is situated at its articular extremity, far removed from the vicinity whence these formations originate. Moreover, in cases of multiple epiphyseal exostoses—even when the toes and fingers are involved—the subungual form of the disease is never met with. Pic has described a skeleton which presented 194 epiphyseal exostoses, many of which were situated on the toes and fingers, but not a single one of these was subungual.

Surgeons commonly ascribe the causation of these growths to various extrinsic factors, such as traumata, tight boots, etc.; but, as I have previously pointed out, the clinical history of cases seldom supports this view. Moreover, it is obvious that whatever part such factors may play, they cannot account for the cartilaginous origin of these tumours, nor for the fact that they so often grow again after they have been cut off. In this connection it is curious to note the oft-repeated allegation, that supernumerary digits may likewise re-form after incomplete removal. In these cases we evidently have to do with growth and development, rather than with products of "irritation" and "inflammation."

These formations differ from most other exostoses and from true tumours—(1) In the unusual frequency with which they arise from their seat of election, viz. the great toe; (2) in the extraordinary constancy of their localisation to the tibial side of the ungual phalanx, near its distal end; and (3) in the structure of the tumour itself, as previously detailed.

In these respects, the subungual exostosis appears to me to bear more resemblance to a teratological anomaly, than to a neoplasm.

Recent researches as to the origin and development of the limbs of vertebrate animals indicate, that we must look to a paddle-like structure with more than five digital rays for the architype of the human foot and hand. Supernumerary digits probably result from the dichotomous division of one or more of the terminal rays at the extremity of a limb. Such reduplication may be partial or complete. From redundant matricular elements thus formed, a more or less perfectly-developed supernumerary digit may evolve; or, in its least complete form, the anomaly may eventuate merely in the formation of a bifid terminal phalanx. In other cases, the redundant matricular elements may undergo more or less complete suppression, only some rudiments being discoverable in the tissues of the part in the adult, or, may be, only in the embryo. On this subject Wiedersheim remarks: "In human embryos of the second month a distinct cartilage is present on the tibial side of the tarsus, and this probably answers to a small bone on the tibial border of the foot of monotremos, American marsupials, edentates, carnivores, rodents, insectivores and monkeys. This most likely corresponds to a redundant first toe (pre-hallux)." Bardeleben maintains that the ancestors of modern mammals were heptadactylous, and that they have lost a digit from the post-axial as well as from the pre-axial side of the foot.

I think the evidence here adduced, is sufficient to warrant me in associating the cartilaginous germ of the subungual exostosis of the great toe, with the rudiment of this lost pre-hallux in its least complete form; and it accords with this, that digital anomalies *per excessum* are of most frequent occurrence in connection with the great toe.

### BIBLIOGRAPHY.

DUPUYTREN. *Leçons Orales de Clin. Chir.*, t. iii., p. 412; see also *Injuries and Diseases of the Bones*, Syd. Soc. transl., 1847, p. 408.

COOPER, ASTLEY. *Surgical Essays*, 1818.

LISTON, R. *Practical Surgery*, 1837, p. 295.

PAGET, JAMES. *Surgical Pathology*, vol. ii., 1853, p. 238.

HUTCHINSON, J. *Lancet*, 1857, ii. 246.

FONTANEL. "Sur l'exostose sous-unguéale de la main," *Thèse de Paris*, 1877.

SOUTHAM, F. A. *Med. Chron.*, 1885–86, iii. 381.

LEBOUC. *Étude clin. et anat. sur quelques cas de tumeurs sous-unguéales.* Paris, 1889.

VARIOT. "Note sur la structure et le dév. de l'exostose sous-unguéale du gros orteil," *Rev. de Chir.*, 1881, i. 480.

WILLIAMS, W. R. "The True Nature of Subungual Exostosis of the Great Toe," *Lancet*, 1890, ii., p. 666.

DE SAINT-GERMAIN. "Exostose sous-unguéale chez les enfants," *Rev. mens. d. mal de l'enf.*, 1884, ii. 316.

REVERDIN. "Exostose de la troisième phalange de l'index," *Rev. méd. de la Suisse Rom.*, 1885, v. 744.

MIRAMOND. "Des Exostoses sous-unguéales," *Thèse de Lyon*, 1894.

PONCET. Duplay and Reclus's *Traité de Chirurgie*, t. ii., 1897, p. 982.

